# Association between quality of life and pregnancy outcomes among infertility patients undergoing assisted reproduction technologies: a prospective cohort study

**DOI:** 10.1080/07853890.2026.2731735

**Published:** 2026-10-14

**Authors:** Pengfei Qu, Yu Zhang, Yiting Li, Kangxin Wang, Qiyue Hu, Lu Gao, Xiaojie Fu, Sirui Liang, Fuyang Zhao, Siyi Ren, Sifan He, Yuan Liu, Yuanyuan Man, Doudou Zhao, Xu Cao, Juanzi Shi

**Affiliations:** a Assisted Reproduction Center, Northwest Women’s and Children’s Hospital, Xi’an, Shaanxi, China; b Department of Epidemiology and Biostatistics, School of Public Health, Xi’an Jiaotong University Health Science Center, Xi’an, China; c Graduate Department, Xi’an Medical University, Xi’an, China; d School of Nursing, Shaanxi University of Chinese Medicine, Xian yang, Shaanxi, China; e The Center of Medical Genetics, Northwest Women’s and Children’s Hospital, Xi’an, Shaanxi Province, China

**Keywords:** Fertility quality of life tool, embryo transfer, cumulative live birth rates, Latent profile analysis, pregnancy outcome

## Abstract

**Background:**

Infertility and its treatment impose significant psychosocial burdens that impair quality of life (QoL). Its impact on pregnancy outcomes after embryo transfer (ET) in assisted reproduction technologies (ART) remains underexplored.

**Objective:**

This study prospectively examined the association between QoL—measured by the FertiQoL instrument—and cumulative live birth rates (CLBRs) in women undergoing ART.

**Methods:**

In this prospective cohort study, 1,233 women initiating IVF/ICSI completed the FertiQoL questionnaire pretreatment. Latent profile analysis (LPA) identified distinct QoL subgroups based on domain scores. Multivariable regression models, adjusted for confounders, assessed associations between QoL profiles/scores and pregnancy outcomes, with CLBRs as the primary endpoint. Smoothed curve fitting and threshold analyses explored nonlinear relationships.

**Results:**

LPA delineated three FertiQoL subgroups: Low (*n* = 278), Medium (*n* = 676), and High (*n* = 279). Multivariate regression revealed that, relative to the Low-QoL group, the Medium-QoL group had significantly higher CLBRs (adjusted odds ratio [OR]: 1.704; 95% CI: 1.217–2.380) and reduced miscarriage risk (adjusted OR: 0.513; 95% CI: 0.304–0.875). Smoothed curve fitting indicated a nonlinear association between total FertiQoL score and CLBRs. Threshold analysis identified a cutoff of 74.306 (log-likelihood ratio test *p* = 0.054); below this value, each one-unit increase in score corresponded to a 2.1% higher CLBR (adjusted OR: 1.021; 95% CI: 1.006–1.036). Bootstrap internal validation confirmed the stability of this cutoff.

**Conclusion:**

QoL was significantly associated with CLBRs following ET. A threshold effect was evident: improvements in QoL below a score of 74.306 were associated with higher live birth rates. These findings underscore the importance of integrating QoL assessment and intervention into infertility care.

## Introduction

Infertility is defined as “the inability to achieve pregnancy after 12 months or more of unprotected regular sexual intercourse” [1]. It significantly impacts the lives of couples [2,3] According to the global infertility epidemiology survey, approximately 50 million people worldwide were affected by infertility in 2010 [4]. In recent years, the number of individuals with infertility has been steadily increasing [5]. Substantial evidence indicates that infertility and its treatment constitute a profoundly stressful life experience, frequently accompanied by significant psychosocial burdens including anxiety, depression, and perceived distress [6–9]. These psychological responses significantly diminish overall life satisfaction and well-being among affected individuals [10–12].

Recognizing the substantial psychosocial burden of infertility, the World Health Organization (WHO) emphasizes quality of life (QoL) as a critical outcome measure, defining it as “an individual’s perception of their position in life within the context of the culture and value systems in which they live, and in relation to their goals, expectations, standards, and concerns” [13]. To standardize the assessment of infertility-specific QoL, the European Society of Human Reproduction and Embryology (ESHRE) and the American Society for Reproductive Medicine (ASRM) collaboratively developed the Fertility Quality of Life (FertiQoL) instrument [14]. FertiQoL has emerged as the gold-standard tool, extensively implemented in global population-based studies to evaluate QoL among individuals experiencing infertility [14–16]. The FertiQoL tool comprises a core module and a selective treatment module. The core module evaluates emotional, mind/body, relational, and social domains, while the selective treatment module assesses the treatment environment and treatment tolerance domains. A higher total FertiQoL score indicates better QoL for patients.

Assisted reproduction technologies (ARTs) are treatments widely used for infertile patients. Research has shown that pregnancy outcomes following embryo transfer (ET) are influenced by factors such as age, body mass index (BMI), emotional distress, and lifestyle [17–22]. However, although QoL has been extensively studied as a core psychosocial indicator among infertility patients, research examining the association between QoL and post-ET pregnancy outcomes remains extremely limited and underexplored. Some studies have found that emotional domain QoL scores were significantly positively correlated with ongoing pregnancy and live birth rates [23,24]. This finding underscores the critical role of the emotional dimension in predicting pregnancy outcomes, yet the comprehensive impact of multi-domain QoL still requires validation in larger samples. Given this gap in the literature and the significance of our cohort, this study aims to prospectively assess the QoL status of infertile patients using the FertiQoL, with a primary focus on analyzing the influence of their QoL scores—including total scores and domain-specific scores—on pregnancy outcomes following ET.

## 2. Materials and methods

### 2.1. Study design and participants

This prospective cohort study included women who received *in vitro* fertilization (IVF) or intracytoplasmic sperm injection (ICSI) treatment at the Center for Assisted Reproductive Technology (ART) of Northwest Women’s and Children’s Hospital in the People’s Republic of China between January and June 2020. Researchers administered the FertiQoL questionnaire using paper-based instruments. Participants voluntarily completed the survey on-site during pre-treatment consultations under research coordinator supervision. Completed questionnaires were secured immediately upon completion. Subsequent pregnancy and neonatal outcomes were prospectively tracked over two years through electronic medical records and structured telephone follow-ups. A total of 1,500 questionnaires were distributed, with 1,435 completed, yielding a 96% recovery rate. Data from 1,233 patients were included in the final statistical analysis. Inclusion criteria were as follows: (1) Confirmed diagnosis aligning with international IVF/ICSI indications per ESHRE/ASRM guidelines, including tubal factor infertility (bilateral occlusion or hydrosalpinx), severe male factor (sperm concentration <5 million/mL or total motility <10%), ovulatory disorders (PCOS or WHO Group I-II anovulation), endometriosis (ASRM Stage III-IV), or unexplained infertility with ≥3 consecutive failed intrauterine insemination cycles [25,26]; (2) Patients with voluntary participation in the study, including signing an informed consent form and complying with ethical standards. Exclusion criteria were as follows: (1) Patients unable to complete the questionnaire independently; (2) Patients with canceled transfers, missed follow-ups, or data loss; (3) Patients who underwent intrauterine insemination; (4) Patients with incomplete questionnaire responses. The flow chart of the study participants is shown in Supplementary Figure S1. This study was approved by the Institutional Review Board of the Northwest Women’s and Children’s Hospital (Assigned Number: 2019-025).

### 2.2. FertiQoL

The FertiQoL was developed through collaboration between the ESHRE and the ASRM, along with Merck-Serono S.A., a leading pharmaceutical company (https://sites.cardiff.ac.uk/fertiqol/). It is the First internationally validated instrument for assessing quality of life among Individuals with fertility issues. The FertiQoL tool comprises a core module and a selective treatment module. The Core Module evaluates four critical domains: the Emotional domain assesses affective states including depression, anxiety, jealousy, and anger; the Mind/Body domain measures physical manifestations such as fatigue and pain alongside cognitive-behavioral impairments exemplified by concentration difficulties, daily activity disruption, and delayed life planning; the Relational domain examines consequences for partner dynamics, sexual functioning, and social engagement; while the Social domain appraises environmental stressors encompassing perceived isolation or support from familial, collegial, and social networks. Complementarily, the Treatment Module addresses intervention-related dimensions through its Treatment Environment component, which quantifies satisfaction with clinical infrastructure, healthcare personnel, and informational resources, and its Treatment Tolerability component, which gauges physical burden, treatment-induced discomfort, and functional interference with daily living [14].

The FertiQoL consists of 36 items, which are used to derive six subscales and three total scores: the total FertiQoL score, the core FertiQoL score, and the treatment FertiQoL score (see detailed information at www.fertiqol.org). The total FertiQoL score represents the combined QoL from both the core and treatment FertiQoL scores. The core FertiQoL score evaluates fertility-related QoL across the emotional, Mind-body, relational, and social subscales, while the treatment FertiQoL score assesses QoL related to the treatment environment and treatment tolerability. The response format for the 36 items is based on a five-point Likert scale. The FertiQoL total and subscale scores are calculated from 0 to 100, with higher scores indicating better quality of life [27].

### 2.3. Data collection

Information for each participant was collected. Trained investigators used structured questionnaires to collect baseline data, including sociodemographic characteristics (age, ethnicity, educational status, occupation, annual income, and BMI), as well as the FertiQoL questionnaire. After two rounds of verification, the questionnaire data were collected and entered into EpiData 3.0 using a dual entry method. Data entry was completed by the project team at the Center for ART of Northwest Women’s and Children’s Hospital in the People’s Republic of China. Any errors detected during the data entry process were corrected through telephone follow-up or review of medical records. Baseline data (type of infertility, infertility factors, infertility duration, antral follicle count (AFC), number of oocytes retrieved, fertilization type, type of transfer, ET stage, number of embryos transferred, and quality of ET) and pregnancy outcomes (cumulative live birth rates [CLBRs], biochemical pregnancy, clinical pregnancy, miscarriage, preterm birth, twin birth, mode of delivery, and birth weight) were retrieved and exported from the medical record system.

### 2.4. Study procedures of ART

Ovarian stimulation in ART typically involved the GnRH agonist long protocol or the GnRH antagonist protocol. The choice of protocol and the starting dose relied on the characteristics of patients and the clinician’s experience. Ovulation was triggered using recombinant human chorionic gonadotropin (HCG) when transvaginal ultrasound revealed at least 2 to 3 dominant follicles measuring 17 mm in diameter. Oocyte retrieval was performed 36 h after the trigger injection, guided by ultrasound. Embryos were cultured individually in 50 ml of sequential medium droplets after conventional IVF or ICSI fertilization. The decision to proceed with IVF or ICSI was based on the semen parameters of the male partner. Oocytes were evaluated for successful insemination 16 to 18 h after IVF or ICSI. The choice between fresh ET or frozen embryo transfer (FET) of 1 to 2 cleavage-stage embryos or blastocysts was made according to the patient’s condition and the clinician’s expertise. Luteal support with progesterone supplementation was provided and maintained until 10 weeks of gestation for patients with a normal pregnancy.

### 2.5. Outcome measures and definitions

The primary outcome measure was the cumulative live birth rate (CLBR). Secondary outcomes included the biochemical pregnancy, clinical pregnancy, miscarriage, preterm birth, twin birth, mode of delivery, and birth weight. Definitions used were as follows: (1) Cumulative live birth: Delivery of at least one live-born infant (≥ 22 weeks gestation) during an ovarian-stimulated fresh or subsequent frozen-thawed cycle. (2) Biochemical pregnancy: Serum β-hCG level ≥ 50 IU/L 14 days after ET. (3) Clinical pregnancy: Ultrasound evidence of one or more gestational sacs in the uterus at 6–8 weeks after ET. (4) Miscarriage: Loss of the clinical pregnancy before 22 weeks of gestation. (5) Preterm birth: Delivery occurring between 22 and 37 weeks of gestation. Clinical outcomes were defined according to the 2017 consensus by the ASRM [28].

### 2.6. Statistical analysis

Latent profile analysis (LPA) was conducted using Mplus 8.3 to identify distinct FertiQoL subgroups. Model fit was evaluated using the Akaike Information Criterion (AIC) and Bayesian Information Criterion (BIC), where smaller values indicate superior fit. Classification accuracy was assessed *via* Entropy (range: 0–1), with higher values reflecting greater precision. The optimal number of latent classes was determined using the Lo-Mendell-Rubin likelihood ratio test (LMRT), where a significant p-value (*p* < 0.05) indicated that the k-class model outperformed the (k-1)-class model. Participants were assigned to classes based on maximum posterior probabilities.

The means and standard deviations of continuous variables are presented as means, while the rates of categorical variables are presented as rates. Continuous variables were analyzed using Analysis of Variance (ANOVA), and categorical variables were assessed using the chi-square test. When the conditions for the chi-square test were not met, the Fisher’s exact test was employed. Pairwise comparisons among multiple groups were conducted using the Bonferroni method. Multiple regression analyses were used to explore the relationship between the total FertiQoL score and CLBRs. The total FertiQoL score was analyzed both as a continuous variable and as categorical variables. Crude and adjusted odds ratios (ORs) with 95% confidence intervals (CIs) were calculated. A generalized additive model was applied for smoothed curve fitting, and threshold effects were assessed to determine the cutoff value of the total FertiQoL score that impacts CLBRs. Bootstrap resampling with 500 iterations was performed to internally validate the optimal cutoff values for CLBRs and cesarean section (Supplementary Table S1, Supplementary Figure S3). We applied a directed acyclic graph (Supplementary Figure S2) combined with previous literature to guide the selection of potential confounders.including the woman’s age, the man’s age, ethnic, educational status, annual income, smoking history, gravidity, parity, BMI, type of infertility, infertility factors, infertility duration, AFC Statistical analyses were performed using EmpowerStats (https://www.empowerstats.net/cn/) and R software (http://www.r-project.org). A *p*-value of <0.05 was considered statistically significant. For pairwise comparisons among multiple groups, a *p*-value of <0.0167 was considered significant to account for multiple testing.

## 3. Results

### 3.1. Potential profile analysis of QoL

This study included 1233 infertility patients who were classified into three distinct subgroups through LPA of FertiQoL dimensions. Models with 2–6 classes were evaluated, with the 3-class solution selected as optimal based on fit indices and practical significance (Table 1). This model demonstrated strong classification accuracy (Entropy = 0.830), statistically significant improvement over the 2-class model (LMRT, *p* < 0.0001), and an acceptable smallest class size (22.5%). Although 4–6 class models yielded lower AIC and BIC values, they showed reduced entropy and included classes with limited clinical utility. The final classification comprised Low FertiQoL (L-FertiQoL, *n* = 278), Medium FertiQoL (M-FertiQoL, *n* = 676), and High FertiQoL (H-FertiQoL, *n* = 279) groups (Supplementary Figure S1). Significant gradients were observed across all FertiQoL domains (Figure 1). The total FertiQoL score and individual module scores for the three groups are presented in Table 2.

**Figure 1.**
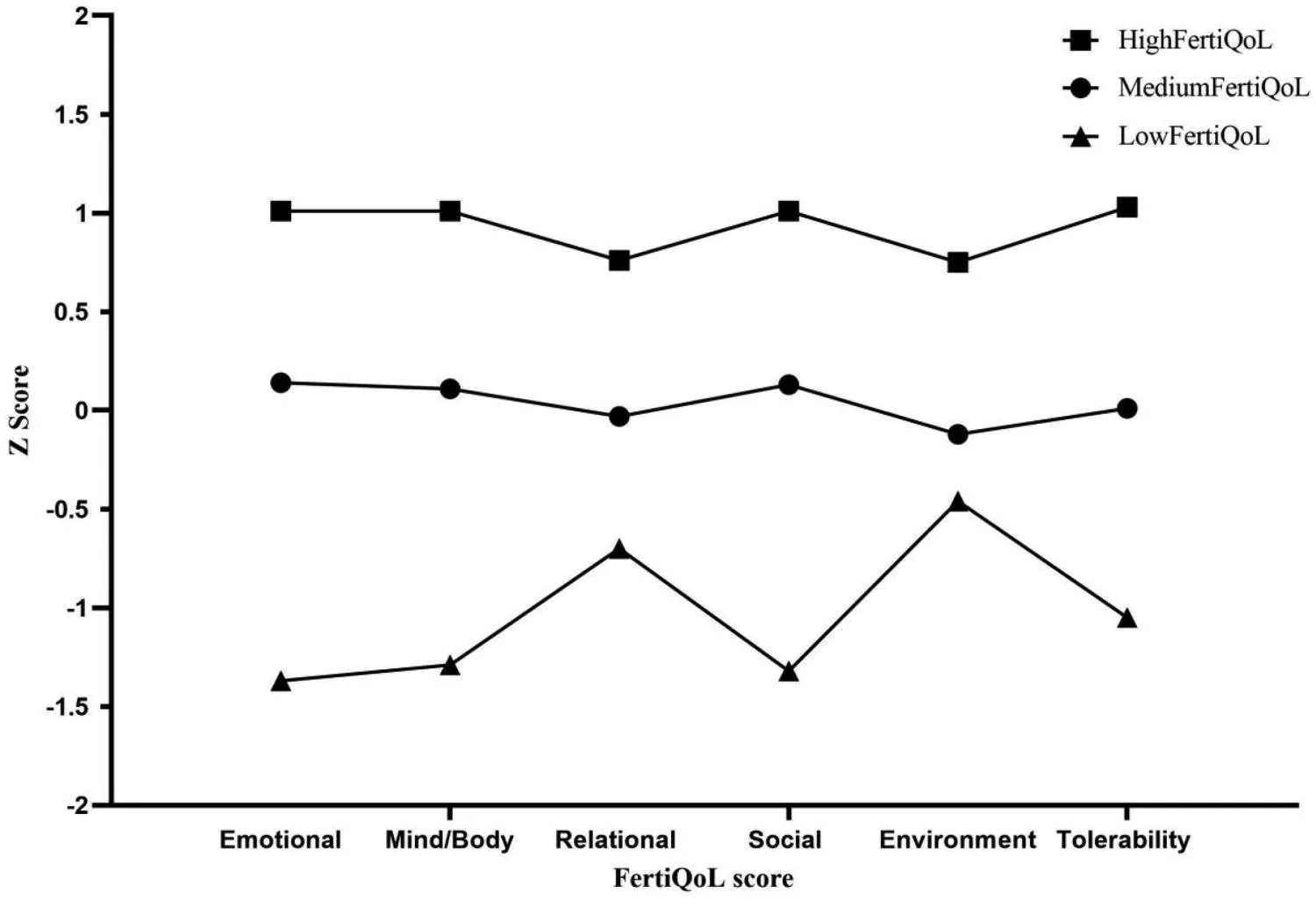
Distribution of characteristics of three potential categories of QoL in infertility patients.

**Table 1.** Comparison of fit indicators for latent profile analysis models with 2 to 6 classes.

N.Class	AIC	BIC	Entropy	LMR	*p*-value of LMRT	Size of smallest class
2-Class	18619.2	18747.1	0.830	2311.2	<0.0001	32.8%
**3-Class**	**17537.0**	**17731.5**	**0.830**	**1058.6**	**<0.0001**	**22.5%**
4-Class	17153.8	17414.8	0.809	390.9	<0.0001	10.5%
5-Class	16883.8	17211.3	0.811	282.8	<0.0001	10.2%
6-Class	9601.4	9995.4	0.815	6981.5	<0.0001	2.5%

*Note*: AIC, Akaike Information Criterion; BIC, Bayesian Information Criterion; LMRT, Lo-Mendell-Rubin Likelihood Ratio Test.

**Table 2.** Comparison of the distribution of FertiQoL score among the three groups.

Characteristics	L-FertiQoL(*n* = 278)	M-FertiQoL(*n* = 676)	H-FertiQoL(*n* = 279)
Total score	49.74 ± 7.76	67.78 ± 4.65	81.43 ± 6.54
Core FertiOoL	46.35 ± 9.40	68.28 ± 5.65	81.88 ± 7.07
Emotional	42.63 ± 13.59	69.15 ± 9.07	84.41 ± 8.78
Mind/Body	52.17 ± 12.51	74.00 ± 8.84	87.95 ± 7.88
Relational	48.32 ± 10.83	57.38 ± 9.66	68.09 ± 16.62
Social	47.90 ± 13.26	70.21 ± 7.86	83.78 ± 7.77
Treatment FertiOoL	55.48 ± 11.63	66.31 ± 8.54	80.82 ± 11.20
Environment	59.85 ± 11.61	64.18 ± 8.94	75.12 ± 15.52
Tolerability	48.92 ± 17.43	69.50 ± 13.39	89.36 ± 11.32

### 3.2. Patient characteristics

Significant differences were observed in several baseline characteristics across the FertiQoL groups. Compared to other groups, the H-FertiQoL group had older male partners (*p* = 0.007), lower proportions of university-educated patients (*p* < 0.001), and higher rates of secondary infertility (*p* = 0.005). Annual income distributions differed significantly (*p* = 0.001), with both the L-FertiQoL and H-FertiQoL groups showing higher proportions of low-income patients (≤50,000 yuan/year) than the M-group. The L-FertiQoL group exhibited lower gravidity and parity, along with fewer fresh embryo transfers. Ethnicity distributions also varied. Other baseline variables did not show significant differences between groups. Details are presented in Table 3.

**Table 3.** Baseline characteristics of infertility patients by FertiQoL subgroup.

Characteristics	L-FertiQoL(*n* = 278)	M-FertiQoL(*n* = 676)	H-FertiQoL(*n* = 279)	*p* value
Age of the woman (years, mean ± SD)	31.06 ± 3.92	30.94 ± 3.60	31.32 ± 4.23	0.600
Age of the man (years, mean ± SD)	32.55 ± 4.37	32.20 ± 4.34	33.29 ± 5.01	0.007
Ethnic, n (%)				0.028*
Han Nationality	276 (99.28)	668 (98.82)	270 (96.77)	
Hui Nationality	1 (0.36)	7 (1.04)	4 (1.43)	
Other Nationality	1 (0.36)	1 (0.15)	5 (1.79)	
Educational status, n(%)				<0.001
Junior high school and below	55 (19.78)	99 (14.64)	72 (25.81)	
Senior high school and Associate degree	128 (46.04)	307 (45.41)	138 (49.46)	
University and beyond	95 (34.17)	270 (39.94)	69 (24.73)	
Occupation, n(%)				0.190
Service	100 (35.97)	196 (28.99)	95 (34.05)	
Worker	14 (5.04)	36 (5.33)	15 (5.38)	
Private owners	19 (6.83)	47 (6.95)	27 (9.68)	
Administrators	35 (12.59)	116 (17.16)	28 (10.04)	
Unemployed person	25 (8.99)	61 (9.02)	21 (7.53)	
Professional specialty	42 (15.11)	122 (18.05)	46 (16.49)	
Other laborers	43 (15.47)	98 (14.50)	47 (16.85)	
Annual income(yuan)				0.001
≤50000	126 (45.32)	210 (31.07)	120 (43.01)	
50000-100000	87 (31.29)	268 (39.64)	88 (31.54)	
100000-200000	40 (14.39)	127 (18.79)	42 (15.05)	
≥200000	25 (8.99)	71 (10.50)	29 (10.39)	
Smoking history, n (%)				0.932*
Non-smoker	273 (98.20)	666 (98.52)	274 (98.21)	
Former smoker	1 (0.36)	3 (0.44)	2 (0.72)	
Current smoker	4 (1.44)	7 (1.04)	3 (1.08)	
Gravidity, n (%)				0.001
0	170 (61.15)	392 (57.87)	131 (46.95)	
1	62 (22.30)	173 (25.59)	83 (29.75)	
2	35 (12.59)	89 (13.17)	43 (15.41)	
≥3	11 (3.96)	21 (3.11)	22 (7.88)	
Parity, n (%)				<0.001
0	255 (91.73)	601 (88.91)	209 (74.91)	
1	21 (7.55)	73 (10.80)	62 (22.22)	
≥2	2 (0.72)	2 (0.30)	8 (2.87)	
BMI (kg/m^2^, mean ± SD)	22.96 ± 3.72	22.70 ± 3.29	23.06 ± 3.55	0.370
<18.5	16 (5.76)	45 (6.66)	20 (7.17)	0.084
18.5–23.9	167 (60.07)	434 (64.20)	149 (53.41)	
24.0–27.9	68 (24.46)	146 (21.60)	81 (29.03)	
≥28	27 (9.71)	51 (7.54)	29 (10.39)	
Type of infertility, n(%)				0.005
Primary	170 (61.15)	394 (58.28)	135 (48.39)	
Secondary	108 (38.85)	282 (41.72)	144 (51.61)	
Infertility factors, n(%)				0.158
Tubal disease	157 (56.47)	368 (54.44)	148 (53.05)	
Ovulatory disorder	54 (19.42)	95 (14.05)	40 (14.34)	
Endometriosis	12 (4.32)	25 (3.70)	8 (2.87)	
Male factor	36 (12.95)	124 (18.34)	58 (20.79)	
Others	19 (6.83)	64 (9.47)	25 (8.96)	
Infertility duration (years, mean ± SD)	3.44 ± 2.47	3.23 ± 2.13	3.52 ± 2.45	0.302
AFC (n, mean ± SD)	13.10 ± 6.79	13.18 ± 6.48	13.19 ± 6.24	0.979
No. of oocyte retrieved (n, mean ± SD)	11.59 ± 7.09	11.43 ± 6.47	11.92 ± 6.13	0.361
Cycle of ET(n, mean ± SD)	1.50 ± 0.73	1.42 ± 0.66	1.38 ± 0.63	0.118
Fertilization type, n (%)				0.890
IVF	212 (76.26)	516 (76.33)	209 (74.91)	
ICSI	66 (23.74)	160 (23.67)	70 (25.09)	
Type of transfer, n(%)				0.003
Fresh-ET	92 (33.09)	296 (43.79)	128 (45.88)	
FET	186 (66.91)	380 (56.21)	151 (54.12)	
Type of embryo transferred, n(%)				0.244
Cleavage stage	85 (30.58)	180 (26.63)	75 (26.88)	
Blastocyst stage	193 (69.42)	496 (73.37)	204 (73.12)	
No. of embryos transferred, n(%)				0.336
1	177 (63.67)	463 (68.49)	190 (68.10)	
2	101 (36.33)	213 (31.51)	89 (31.90)	
Good quality embryo transfer, n(%)	155 (55.76)	385 (56.95)	161 (57.71)	0.895

*Note*: *Fisher’s exact test.

Data are presented as n (%) or mean ± SD. SD, standard deviation; BMI: body mass index; AFC: antral follicle count; IVF: *in vitro* fertilization; ICSI: intracytoplasmic sperm injection; ET: embryos transfer; FET: frozen-thawed embryo transfer; Good quality cleavage-stage embryos were defined as those derived from normally zygote with an embryonic cell number of 7–9 and a fragmentation level of less than 10% on day 3 after fertilization; high-quality blastocysts were defined as those with a score of better than 4CC.

### 3.3. Primary and secondary outcomes of different groups

Significant differences in CLBRs were observed across FertiQoL groups (*p* = 0.002). The M-FertiQoL group demonstrated the highest CLBR (77.96%), significantly exceeding the L-FertiQoL group (67.63%). Miscarriage rates also differed significantly (*p* = 0.016), with the M-FertiQoL group showing the lowest rate (7.36%). Other pregnancy outcomes, including the biochemical pregnancy, clinical pregnancy, preterm birth, twin birth, mode of delivery, and birth weight did not show statistically significant differences across the three groups. The detailed results are presented in Table 4.

**Table 4.** Comparison of pregnancy outcomes across FertiQoL profile groups.

Pregnancy outcomes	L-FertiQoL	M-FertiQoL	H-FertiQoL	*p* value
Cumulative live birth, n(%)	188 (67.63)	527 (77.96)	200 (71.68)	0.002
Biochemical pregnancy, n(%)	237 (85.25)	587 (86.83)	237 (84.95)	0.678
Clinical pregnancy, n(%)	226 (81.29)	571 (84.47)	233 (83.51)	0.486
Miscarriage, n(%)	30 (13.27)	42 (7.36)	28 (12.02)	0.016
Preterm birth, n(%)	26 (13.83)	72 (13.66)	24 (12.00)	0.820
Twin birth, n(%)	20 (10.64)	50 (9.49)	17 (8.50)	0.772
Mode of delivery, n(%)				0.076
Natural labor	63 (33.51)	134 (25.43)	61 (30.50)	
Cesarean section	125 (66.49)	393 (74.57)	139 (69.50)	
Birth weight (g, mean ± SD)	3262 ± 567	3218 ± 554	3245 ± 580	0.610

*Note*: Data are presented as *n* (%) or mean ± SD. SD, standard deviation.

### 3.4. Multivariable regression analysis of FertiQoL profiles and pregnancy outcomes

In multivariable regression analyses, the total FertiQoL score was positively associated with CLBRs after adjustment (adjusted OR: 1.012, 95% CI: 1.000–1.023, *p* = 0.044), while no significant associations were observed between continuous FertiQoL scores and the remaining pregnancy outcomes (*p* > 0.05). When analyzed categorically, the M-FertiQoL group exhibited significantly higher CLBRs (adjusted OR: 1.704, 95% CI: 1.217–2.380) and lower miscarriage risk (adjusted OR: 0.513, 95% CI: 0.304–0.875) compared to the L-FertiQoL group. The medium group also demonstrated increased cesarean delivery odds (adjusted OR: 1.728, 95% CI: 1.168–2.548). No significant associations were found for biochemical pregnancy, clinical pregnancy, preterm birth, twin birth, or birth weight across FertiQoL profiles. The detailed results are presented in Table 5.

**Table 5.** Multivariable regression analysis of FertiQoL score on pregnancy outcomes.

Pregnancy outcomes	Group	Crude model β/OR (95% CI)	*p* value	Adjusted model* β/OR (95% CI)	*p* value
Cumulative live birth	Total score	1.008 (0.998, 1.019)	0.123	1.012 (1.000, 1.023)	0.044
	L-FertiQoL	1.0		1.0	
	M-FertiQoL	1.693 (1.242, 2.309)	0.001	1.704 (1.217, 2.380)	0.002
	H-FertiQoL	1.212 (0.844, 1.740)	0.298	1.323 (0.889, 1.975)	0.169
	P trend	0.276		0.142	
Biochemical pregnancy	Total score	1.001 (0.988, 1.014)	0.894	1.004 (0.990, 1.018)	0.600
	L-FertiQoL	1.0		1.0	
	M-FertiQoL	1.141 (0.765, 1.701)	0.518	1.106 (0.715, 1.688)	0.643
	H-FertiQoL	0.976 (0.612, 1.556)	0.919	1.024 (0.615, 1.705)	0.928
	P trend	0.916		0.925	
Clinical pregnancy	Total score	1.007 (0.995, 1.019)	0.279	1.012 (0.999, 1.025)	0.080
	L-FertiQoL	1.0		1.0	
	M-FertiQoL	1.251 (0.867, 1.805)	0.230	1.205 (0.810, 1.778)	0.351
	H-FertiQoL	1.165 (0.753, 1.804)	0.492	1.333 (0.827, 2.156)	0.239
	P trend	0.481		0.235	
Miscarriage	Total score	0.997 (0.980, 1.014)	0.705	0.996 (0.979, 1.015)	0.685
	L-FertiQoL	1.0		1.0	
	M-FertiQoL	0.519 (0.316, 0.852)	0.010	0.513 (0.304, 0.875)	0.013
	H-FertiQoL	0.892 (0.514, 1.548)	0.685	0.877 (0.479, 1.599)	0.668
	P trend	0.673		0.607	
Preterm birth	Total score	0.999 (0.983, 1.015)	0.876	0.999 (0.982, 1.016)	0.885
	L-FertiQoL	1.0		1.0	
	M-FertiQoL	0.986 (0.608, 1.598)	0.954	1.090 (0.663, 1.846)	0.740
	H-FertiQoL	0.850 (0.469, 1.540)	0.591	0.831 (0.440, 1.564)	0.564
	P trend	0.591		0.563	
Twin birth	Total score	0.987 (0.969, 1.005)	0.150	0.989 (0.970, 1.008)	0.233
	L-FertiQoL	1.0		1.0	
	M-FertiQoL	0.881 (0.509, 1.522)	0.649	0.986 (0.563, 1.793)	0.962
	H-FertiQoL	0.780 (0.395, 1.540)	0.474	0.826 (0.397, 1.702)	0.604
	P trend	0.474		0.610	
Cesarean section	Total score	1.007 (0.995, 1.020)	0.231	1.008 (0.995, 1.021)	0.252
	L-FertiQoL	1.0		1.0	
	M-FertiQoL	1.478 (1.030, 2.120)	0.034	1.728 (1.168, 2.548)	0.006
	H-FertiQoL	1.148 (0.749, 1.760)	0.525	1.212 (0.757, 1.942)	0.423
	P trend	0.545		0.412	
Birth weight	Total score	−1.321 (−4.391, 1.749)	0.399	−1.394 (−4.575, 1.788)	0.390
	L-FertiQoL	1.0		1.0	
	M-FertiQoL	−44.652 (−138.358, 49.055)	0.351	−62.747 (−158.899, 33.406)	0.201
	H-FertiQoL	−17.612 (−129.665, 94.441)	0.758	−16.757 (−133.319, 99.804)	0.778
	P trend	0.775		0.784	

*Note*: *Adjusted for age of the woman, age of the man, ethnic, educational status, annual income, smoking history, gravidity, parity, BMI, type of infertility, infertility factors, infertility duration, AFC. OR, odds ratio; CI, confidence interval.

Analysis of FertiQoL subscales revealed that higher Mind/Body scores were significantly associated with higher CLBRs (adjusted OR: 1.013, 95% CI: 1.004–1.022) and higher clinical pregnancy rates (adjusted OR: 1.012, 95% CI: 1.002–1.022). Similarly, higher Social scores were significantly associated with higher CLBRs (adjusted OR: 1.010, 95% CI: 1.001–1.019) and higher clinical pregnancy rates (adjusted OR: 1.011, 95% CI: 1.000–1.021). Higher Relational scores were associated with lower twin birth risk (adjusted OR: 0.971, 95% CI: 0.952–0.989). No significant associations were observed between any subscales and biochemical pregnancy, miscarriage, preterm birth, cesarean section, or birth weight outcomes after adjustment. Details are presented in Table 6.

**Table 6.** Multivariable regression analysis of FertiQoL subscales and pregnancy outcomes.

Pregnancy outcomes	Group	Crude model OR (95% CI)	*p* value	Adjusted model* OR (95% CI)	*p* value
Cumulative live birth	Emotional	1.005 (0.997, 1.012)	0.219	1.006 (0.998, 1.014)	0.152
	Mind/Body	1.011 (1.003, 1.019)	0.008	1.013 (1.004, 1.022)	0.004
	Relational	1.000 (0.991, 1.010)	0.946	1.002 (0.992, 1.012)	0.748
	Social	1.006 (0.998, 1.014)	0.165	1.010 (1.001, 1.019)	0.034
	Environment	0.997 (0.987, 1.007)	0.584	1.001 (0.990, 1.012)	0.880
	Tolerability	1.004 (0.998, 1.011)	0.206	1.005 (0.988, 1.012)	0.166
Biochemical pregnancy	Emotional	0.999 (0.990, 1.008)	0.833	1.000 (0.991, 1.010)	0.928
	Mind/Body	1.007 (0.997, 1.017)	0.179	1.008 (0.997, 1.019)	0.167
	Relational	0.997 (0.985, 1.009)	0.621	0.999 (0.987, 1.012)	0.928
	Social	1.001 (0.991, 1.011)	0.898	1.003 (0.992, 1.014)	0.578
	Environment	0.993 (0.980, 1.005)	0.266	0.996 (0.983, 1.010)	0.593
	Tolerability	1.002 (0.993, 1.010)	0.720	1.003 (0.994, 1.011)	0.567
Clinical pregnancy	Emotional	1.004 (0.996, 1.013)	0.318	1.077 (0.998, 1.016)	0.131
	Mind/Body	1.010 (1.001, 1.020)	0.039	1.012 (1.002, 1.022)	0.023
	Relational	0.998 (0.987, 1.010)	0.780	1.002 (0.990, 1.014)	0.752
	Social	1.007 (0.997, 1.016)	0.163	1.011 (1.000, 1.021)	0.042
	Environment	0.993 (0.982, 1.005)	0.261	0.998 (0.986, 1.011)	0.800
	Tolerability	1.004 (0.996, 1.012)	0.330	1.006 (0.998, 1.014)	0.145
Miscarriage	Emotional	1.002 (0.990, 1.014)	0.766	1.003 (0.991, 1.016)	0.619
	Mind/Body	0.992 (0.979, 1.005)	0.206	0.991 (0.977, 1.004)	0.179
	Relational	0.995 (0.980, 1.011)	0.538	0.995 (0.978, 1.011)	0.555
	Social	1.001 (0.987, 1.014)	0.943	0.997 (0.983, 1.012)	0.732
	Environment	1.000 (0.984, 1.017)	0.973	1.000 (0.983, 1.017)	0.982
	Tolerability	1.000 (0.990, 1.011)	0.932	1.002 (0.991, 1.014)	0.708
Preterm birth	Emotional	0.997 (0.986, 1.008)	0.627	0.998 (0.987, 1.010)	0.762
	Mind/Body	0.997 (0.985, 1.010)	0.658	0.998 (0.985, 1.011)	0.720
	Relational	0.994 (0.979, 1.008)	0.379	0.991 (0.976, 1.005)	0.211
	Social	0.993 (0.981, 1.006)	0.276	0.994 (0.982, 1.008)	0.402
	Environment	1.010 (0.995, 1.026)	0.190	1.009 (0.992, 1.025)	0.287
	Tolerability	1.008 (0.997, 1.018)	0.141	1.007 (0.997, 1.018)	0.175
Twin birth	Emotional	0.993 (0.980, 1.006)	0.272	0.995 (0.982, 1.009)	0.471
	Mind/Body	0.997 (0.983, 1.012)	0.689	1.002 (0.987, 1.017)	0.840
	Relational	0.974 (0.956, 0.992)	0.005	0.971 (0.952, 0.989)	0.002
	Social	0.989 (0.975, 1.004)	0.141	0.992 (0.977, 1.007)	0.281
	Environment	0.993 (0.975, 1.012)	0.462	0.988 (0.969, 1.008)	0.246
	Tolerability	1.001 (0.989, 1.013)	0.858	1.002 (0.990, 1.014)	0.719
Cesarean section	Emotional	1.005 (0.996, 1.013)	0.289	1.006 (0.997, 1.015)	0.202
	Mind/Body	1.004 (0.995, 1.014)	0.361	1.007 (0.997, 1.017)	0.177
	Relational	1.001 (0.991, 1.012)	0.847	0.999 (0.987, 1.010)	0.813
	Social	1.002 (0.992, 1.012)	0.705	1.003 (0.993, 1.014)	0.526
	Environment	1.007 (0.995, 1.019)	0.259	1.004 (0.991, 1.018)	0.521
	Tolerability	1.009 (1.002, 1.017)	0.016	1.008 (1.000, 1.016)	0.063
Birth weight	Emotional	−1.189 (−3.340, 0.963)	0.279	−1.421 (−3.648, 0.805)	0.211
	Mind/Body	0.297 (−2.121, 2.715)	0.810	0.081 (−2.391, 2.533)	0.949
	Relational	−1.379 (−4.044, 1.287)	0.311	−0.792 (−3.546, 1.963)	0.573
	Social	−1.652 (−4.105, 0.801)	0.187	−1.949 (−4.494, 0.596)	0.133
	Environment	−0.543 (−3.552, 2.466)	0.724	−0.294 (−3.438, 2.850)	0.854
	Tolerability	−0.735 (−2.650, 1.180)	0.452	−0.759 (−2.728, 1.210)	0.450

*Note*: Core subscales: Emotional, Mind/Body, Relational, Social. Treatment subscales: Environment, Tolerability.

*Adjusted for age of the woman, age of the man, ethnic, educational status, annual income, smoking history, gravidity, parity, BMI, type of infertility, infertility factors, infertility duration, AFC. OR, odds ratio; CI, confidence interval.

### 3.5. Results of generalized additive model

Piecewise linear regression revealed significant threshold effects for two outcomes (Table 7, Figure 2). For CLBRs, a cutoff of 74.306 was identified (log-likelihood ratio test *p* = 0.054 for model fit). Below this threshold, each unit increase in FertiQoL score was associated with 2.1% higher CLBRs (adjusted OR: 1.021, 95% CI: 1.006–1.036), while no association existed above it (adjusted OR: 0.980, 95% CI: 0.947–1.014). Bootstrap internal validation with 500 resampling iterations confirmed the stability of this cutoff (Supplementary Table S1, Supplementary Figure S3). Similarly, for cesarean section, a cutoff of 62.5 was significant (log-likelihood ratio test *p* = 0.039 for model fit), with scores below this threshold showing 3.2% increased odds per unit (adjusted OR: 1.032, 95% CI: 1.005–1.060). No significant threshold effects were detected for biochemical pregnancy, clinical pregnancy, miscarriage, preterm birth, twin birth, or birth weight.

**Figure 2.**
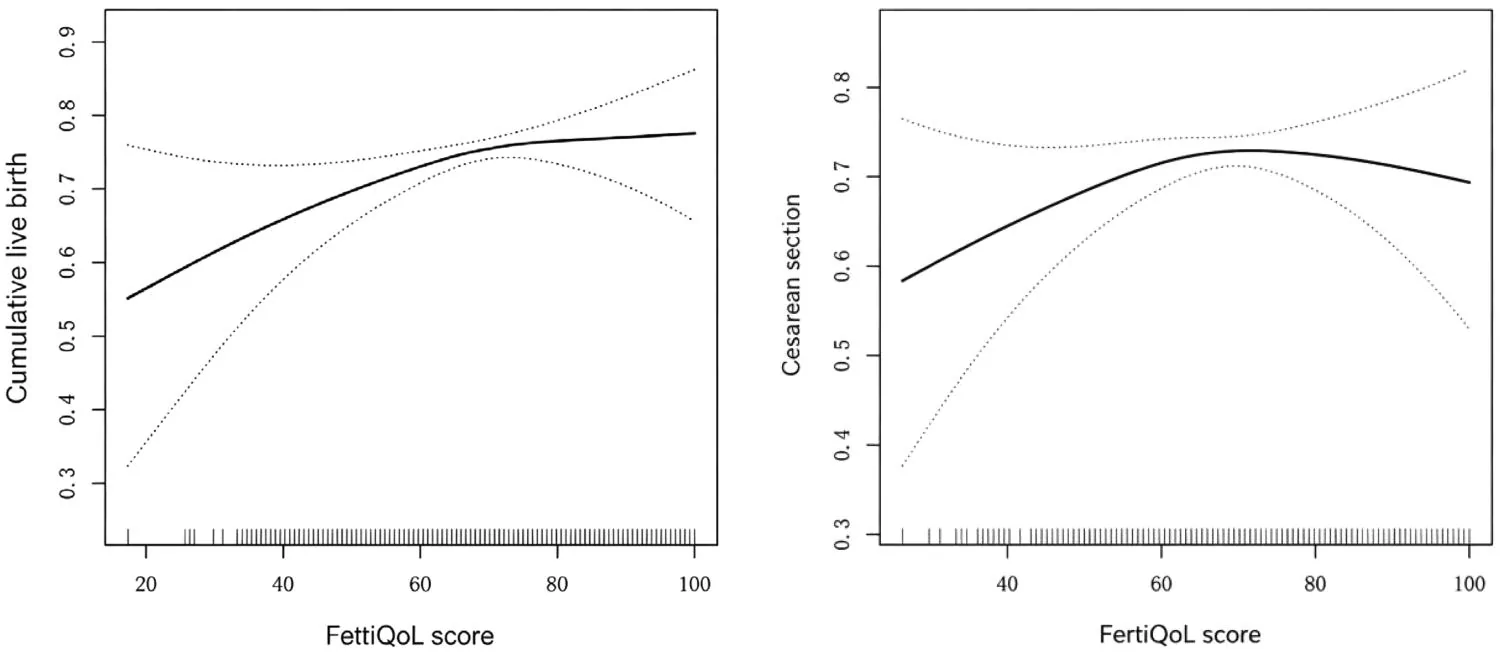
Threshold effect of total FertiQoL score on cumulative live birth rates and cesarean section. *Note*: Adjusted for age of the woman, age of the man, ethnic, educational status, annual income, smoking history, gravidity, parity, BMI, type of infertility, infertility factors, infertility duration, AFC.

**Table 7.** Threshold effects of FertiQoL scores on pregnancy outcomes using piecewise linear regression.

	Outcome	Crude model OR (95% CI)	*p* value	Adjusted model* OR (95% CI)	*p* value
Cumulative live birth	One-line linear regression model	1.008 (0.998, 1.019)	0.123	1.012 (1.001, 1.023)	0.039
	cutoff value (K)	73.611		74.306	
	≤K	1.018 (1.005, 1.032)	0.008	1.021 (1.006, 1.036)	0.005
	>K	0.976 (0.948, 1.006)	0.111	0.980 (0.947, 1.014)	0.237
	Log-likelihood ratio test	0.027		0.054	
Biochemical pregnancy	One-line linear regression model	1.001 (0.988, 1.014)	0.894	1.004 (0.989, 1.018)	0.620
	cutoff value (K)	77.778		84.722	
	≤K	1.007 (0.991, 1.023)	0.397	1.008 (0.992, 1.024)	0.335
	>K	0.971 (0.927, 1.017)	0.214	0.952 (0.874, 1.038)	0.268
	Log-likelihood ratio test	0.202		0.246	
Clinical pregnancy	One-line linear regression model	1.007 (0.995, 1.019)	0.279	1.011 (0.998, 1.025)	0.088
	cutoff value (K)	74.306		77.083	
	≤K	1.014 (0.999, 1.030)	0.072	1.017 (1.001, 1.033)	0.036
	>K	0.980 (0.946, 1.016)	0.280	0.984 (0.939, 1.031)	0.492
	Log-likelihood ratio test	0.140		0.240	
Miscarriage	One-line linear regression model	0.997 (0.980, 1.014)	0.705	0.994 (0.976, 1.013)	0.544
	cutoff value (K)	84.722		84.722	
	≤K	1.000 (0.982, 1.019)	0.980	0.998 (0.979, 1.019)	0.870
	>K	0.928 (0.782, 1.100)	0.389	0.902 (0.737, 1.104)	0.318
	Log-likelihood ratio test	0.355		0.280	
Preterm birth	One-line linear regression model	0.999 (0.983, 1.015)	0.876	0.999 (0.982, 1.016)	0.893
	cutoff value (K)	81.944		81.944	
	≤K	0.992 (0.975, 1.010)	0.407	0.993 (0.975, 1.012)	0.481
	>K	1.052 (0.979, 1.131)	0.169	1.045 (0.970, 1.125)	0.250
	Log-likelihood ratio test	0.173		0.247	
Twin birth	One-line linear regression model	0.987 (0.969, 1.005)	0.150	0.989 (0.970, 1.008)	0.257
	cutoff value (K)	53.472		76.389	
	≤K	0.957 (0.903, 1.014)	0.136	0.995 (0.972, 1.018)	0.666
	>K	0.996 (0.972, 1.021)	0.760	0.956 (0.882, 1.037)	0.280
	Log-likelihood ratio test	0.293		0.371	
Cesarean section	One-line linear regression model	1.007 (0.995, 1.020)	0.231	1.007 (0.993, 1.020)	0.323
	cutoff value (K)	70.833		62.500	
	≤K	1.018 (1.001, 1.036)	0.037	1.032 (1.005, 1.060)	0.022
	>K	0.984 (0.956, 1.013)	0.273	0.988 (0.966, 1.010)	0.270
	Log-likelihood ratio test	0.086		0.039	
Birth weight	One-line linear regression model	−1.321 (−4.391, 1.749)	0.399	−1.347 (−4.545, 1.852)	0.410
	cutoff value (K)	84.722		61.111	
	≤K	−0.520 (−3.950, 2.911)	0.767	−4.260 (−11.631, 3.112)	0.258
	>K	−12.140 (−33.028, 8.747)	0.255	0.433 (−4.734, 5.600)	0.870
	Log-likelihood ratio test	0.304		0.383	

*Note*: *Adjusted for age of the woman, age of the man, ethnic, educational status, annual income, smoking history, gravidity, parity, BMI, type of infertility, infertility factors, infertility duration, AFC. OR, odds ratio; CI, confidence interval; K, optimal cutoff value determined by likelihood ratio test.

## 4. Comment

### 4.1. Main findings

This prospective cohort study employed LPA to classify 1,233 infertility patients into three distinct FertiQoL subgroups (Low, Medium, High). Our primary finding indicates that women with medium FertiQoL scores exhibited significantly higher CLBRs and lower miscarriage risk compared to the Low-FertiQoL group, even after rigorous adjustment for clinical confounders. Crucially, threshold analysis identified 74.306 as the cutoff for the total FertiQoL score, below which each unit increase was associated with significantly higher CLBRs (2.1% per unit). These results reveal a non-linear relationship between QoL and live birth outcomes, suggesting that optimal pregnancy success is associated with moderate QoL levels.

There has been limited research on the relationship between QoL in infertility patients and pregnancy outcomes after ET. Santoro et al. investigated the QoL in patients with unexplained infertility (UI) and polycystic ovary syndrome (PCOS) and found that the score of the emotional subscale of the FertiQoL was positively correlated with live birth rates and conception chances in PCOS patients, though the significance was unclear in UI patients [29]. The inconsistent results led Santoro et al. to suggest that QoL might not be a crucial predictor of pregnancy outcomes [29]. In contrast, Yoko Urata et al. found that in women undergoing IVF treatment, a higher FertiQoL treatment module score was associated with a higher rate of positive gestational sac detection after embryo transfer [30]. This study also found a significant positive correlation between the total FertiQoL score and CLBRs. Given that low-QoL in infertility patients is often associated with severe emotional distress, including stress, anxiety, and depression [31–33]. Notably, our results align with longitudinal studies by Wu et al. where higher emotional domain scores predicted increased ongoing pregnancy and live birth rates in IVF patients [24], underscoring the critical role of psychological well-being.

This study found that while the M-FertiQoL group exhibited significantly higher CLBRs and lower miscarriage risk compared to the L-FertiQoL group, no such advantages were observed in the H-FertiQoL group despite their superior FertiQoL scores. We propose two mechanistic explanations for this phenomenon. First, patients in the M-FertiQoL range may experience moderate stress levels that enhance physiological adaptation (e.g. optimized HPA axis activity promoting oocyte maturation [34], whereas L-FertiQoL individuals endure overwhelming distress that suppresses reproductive function through cortisol dysregulation [9]. Conversely, H-FertiQoL patients’ minimized distress may reflect avoidant coping strategies—such as disengagement from treatment realities—potentially compromising treatment adherence or self-advocacy. Second, the M-FertiQoL group’s relatively higher socioeconomic status likely facilitates resource access (e.g. timely appointments, nutritional support), while their balanced psychological engagement promotes treatment compliance [6,7,35]. In contrast, H-FertiQoL patients’ heightened sense of control may lead to underestimation of medical risks, mirroring findings that extreme optimism correlates with poorer ART adherence [36].

### 4.2. Biological plausibility

Emotional distress, such as stress, pain, and anxiety, has been shown to impact pregnancy outcomes following ET [18,31,32], and biomarkers of stress, such as salivary α-amylase, are negatively associated with fertility [33]. As shown in network analyses of Chinese infertility patients, symptoms like “social withdrawal” and “self-devaluation” strongly correlate with reduced FertiQoL scores [37]. Previous studies suggest that negative emotions may activate the hypothalamic-pituitary-adrenal (HPA) axis [38], leading to the release of stress hormones, such as cortisol, which inhibit the reproductive system. The activation of the HPA axis could impair estradiol production, damage granulosa cells, disrupt follicular maturation, and reduce the number of oocytes [34], ultimately affecting pregnancy outcomes. Therefore, this study suggests that emotional distress may contribute to lower CLBRs in patients with poorer QoL. Our threshold effect suggests that mitigating distress below this critical point may optimize physiological conditions for pregnancy. Furthermore, controlled ovarian stimulation used in ART is associated with the risk of ovarian hyperstimulation syndrome (OHSS), a serious iatrogenic complication, and oocyte vitrification has been proposed as an effective strategy for fertility preservation that may also reduce this risk [39,40]. Nutraceutical supplementation with inositols has also been reported to exert beneficial biological effects on oocyte quality and reproductive outcomes in infertile patients [41].

### 4.3. Strengths and limitations

This study had several strengths. Firstly, the questionnaire was administered to patients before ART treatments, which might effectively reduce recall bias. Secondly, the study included a relatively large sample size, accounted for multiple confounding factors, and employed both continuous and categorical analyses of the total FertiQoL score. Smoothed curve fitting and threshold effect analyses were used to determine the cutoff value for CLBRs, enhancing the accuracy of the results. Third, using data directly from the clinic minimized observational bias.

However, several limitations warrant consideration. First, despite adjusting for numerous confounders through multivariable regression analyses, unaccounted factors—including lifestyle behaviors (e.g. sleep patterns [22] and psychosocial confounders—may introduce residual bias. Notably, our exclusion criteria omitted patients with diagnosed mental disorders (e.g. major depression or anxiety disorders) or those experiencing major traumatic life events (e.g. bereavement, job loss, or recent failed ART cycles/pregnancy loss) within 6 months preceding QoL assessment. These factors are established determinants of psychological well-being and QoL metrics, potentially confounding observed associations. Second, while we adjusted for broad infertility etiologies (e.g. tubal, male, ovulatory), granular data regarding prognostically significant diagnostic specifics were unavailable (e.g. endometriosis staging, uterine pathology severity). Additionally, prior ART cycle history—including the number of failed embryo transfers—was not documented, despite its known influence on cumulative live birth rates and potential interaction with QoL. Third, persistent baseline differences between FertiQoL groups (e.g. male partner age, education level, infertility type, income) after statistical adjustment raise concerns about residual confounding, potentially affecting association interpretability. Finally, the exclusive focus on pregnancy outcomes precluded examination of neonatal/perinatal outcomes or pregnancy complications. As a single-center investigation, generalizability requires validation across diverse clinical settings.

## Supplementary Material

Supplementary file.docx

## Data Availability

The data are available from the corresponding author upon reasonable request.

## Clinical trial registration

Not applicable (retrospective observational study).

## Presentation

Not applicable/not previously presented.
