# Supplementary material for: Association between quality of life and pregnancy outcomes among infertility patients undergoing assisted reproduction technologies: a prospective cohort study
Source: Ann Med. 2026 Oct 14;58(1):2731735. doi: 10.1080/07853890.2026.2731735 (PMC13670262; doi:10.1080/07853890.2026.2731735)
Supplement: Supplementary file 1 [file IANN_A_2731735_SM9108.docx]

# Supplementary Materials

**Supplementary Figure S1 | Flow chart of study cohort.**

Note: L-FertiQoL, Low FertiQoL; M-FertiQoL, Medium FertiQoL; H-FertiQoL, High FertiQoL.

**Supplementary Table S1 | Bootstrap internal validation of the optimal cutoffs for FertiQoL total score on cumulative live birth and cesarean section.**

| Outcome | Cutoff (K) | Bootstrap median OR (≤K) | Bootstrap 95% CI | OR > 1 (%) | Convergence |
| --- | --- | --- | --- | --- | --- |
| Cumulative live birth | 74.306 | 1.022 | 1.006–1.036 | 99.4 | 500/500 |
| Cesarean section | 62.500 | 1.032 | 1.006–1.064 | 99.4 | 499/500 |

Note: Bootstrap validation was performed using 500 resampling iterations with replacement. Models were adjusted for maternal age, paternal age, ethnicity, education, income, smoking, gravidity, parity, BMI, infertility type, infertility factors, infertility duration, and AFC. ORs and 95% CIs were derived from the percentile method. Proportion indicates the percentage of bootstrap iterations in which the OR for scores ≤ the cutoff exceeded 1. Convergence rate indicates the number of successful bootstrap iterations out of 500.

**Supplementary Figure S2 | Directed Acyclic Graph for the associations between FertiQoL score and pregnancy outcome.**

Note: BMI: body mass index; AFC: antral follicle count; ET: embryo transfer. Minimally sufficient adjustment set: age of the woman, age of the man, ethnic, educational status, annual income, smoking history, gravidity, parity, BMI, type of infertility, infertility factors, infertility duration, AFC.

**Supplementary Figure S3 | Bootstrap validation of threshold effects.**

Bootstrap distribution of the odds ratio (OR) for FertiQoL total score ≤ the optimal cutoff, based on 500 resampling iterations. The red dashed line indicates OR = 1; the green solid line indicates the bootstrap median OR. These results confirm that the observed threshold effects are robust and not attributable to overfitting.
